# Supplementary material for: An immuno-enrichment free, validated quantification of tau protein in human CSF by LC-MS/MS
Source: PLoS One. 2022 Jun 2;17(6):e0269157. doi: 10.1371/journal.pone.0269157 (PMC9162344; doi:10.1371/journal.pone.0269157)
Supplement: S1 Table — (DOCX) [file pone.0269157.s001.docx]

**S1 Table** Description of CSF samples from persons with Alzheimer’s Disease used in this study.

| AD CSF Sample No. | Age | Gender | Race | Total CSF Tau Concentration (pg/mL) |
| --- | --- | --- | --- | --- |
| 1 | 77 | Male | Caucasian | 1,089 |
| 2 | 63 | Male | Caucasian | 1,265 |
| 3 | 72 | Male | Hispanic | 1,318 |
| 4 | 65 | Male | Hispanic | 715 |
| 5 | 64 | Male | Caucasian | 697 |
| 6 | 63 | Female | Caucasian | 361 |
| 7 | 60 | Female | African American | 366 |
| 8 | 73 | Male | Asian | 1008 |
| 9 | 60 | Female | Asian | 899 |
| 10 | 67 | Female | Hispanic | 718 |
| 11 | 69 | Female | Hispanic | 356 |

The Total CSF Tau Concentration was assayed by Precision Med, Inc. using the Total Tau MSD assay (MesoScale).
