# Supplementary material for: An immuno-enrichment free, validated quantification of tau protein in human CSF by LC-MS/MS
Source: PLoS One. 2022 Jun 2;17(6):e0269157. doi: 10.1371/journal.pone.0269157 (PMC9162344; doi:10.1371/journal.pone.0269157)
Supplement: S2 Table — (DOCX) [file pone.0269157.s002.docx]

**S2 Table.** Mass Spectrometry parameters on the Thermo Q Exactive for Pre-Validation experiments using nanoLC-MS/MS.

| Peptide | Amino Acid Sequence | Precursor  m/z | Charge  (z) | Acquisition Time Start  (min) | Acquisition Time Stop  (min) | HCD Collision Energy  (%) | Injection Time  (ms) | Transitions Analyzed |
| --- | --- | --- | --- | --- | --- | --- | --- | --- |
| 181-190 | TPPSSGEPPK | 498.75 | 2 | 5 | 30 | 25 | 50 | y8+, y7+, y9++ |
|  |  | 504.24 | 2 | 5 | 30 | 25 | 50 |  |
| 260-267 | IGSTENLK | 431.24 | 2 | 5 | 30 | 20 | 50 | y7+,y6+,y5+, y4+ |
|  |  | 436.22 | 2 | 5 | 30 | 20 | 50 |  |
| 396-406 | SPVVSGDTSPR | 551.28 | 2 | 5 | 30 | 25 | 50 | y9+, y8+, y7+, y6+ |
|  |  | 558.26 | 2 | 5 | 30 | 25 | 50 |  |
| 25-44 | DQGGYTMHQDQEGDTDAGLK | 722.64 | 3 | 26 | 37 | 20 | 50 | y18++,y17++, y16++ |
|  |  | 731.28 | 3 | 26 | 37 | 20 | 50 |  |
| 354-369 | IGSLDNITHVPGGGNK | 526.95 | 3 | 26 | 37 | 20 | 50 | y6+, y15++, y14++, y12++ |
|  |  | 533.92 | 3 | 26 | 37 | 20 | 50 |  |
