# Supplementary material for: An immuno-enrichment free, validated quantification of tau protein in human CSF by LC-MS/MS
Source: PLoS One. 2022 Jun 2;17(6):e0269157. doi: 10.1371/journal.pone.0269157 (PMC9162344; doi:10.1371/journal.pone.0269157)
Supplement: S4 Table — (DOCX) [file pone.0269157.s004.docx]

**S4 Table.** Optimization of Perchloric Acid Precipitation conditions.

| Peptide | 2.5% LLOQ  (pg/mL) | Mean Bias  (\|% RE\|) | 1% PCA LLOQ  (pg/mL) | Mean Bias  (\|% RE\|) |
| --- | --- | --- | --- | --- |
| 25-44 | 1400 | 6 | 700 | 3 |
| 181-190 | 700 | 13 | 350 | 20 |
| 260-267 | 350 | 4 | 350 | 25 |
| 354-369 | 700 | 3 | 175 | 8 |
| 396-406 | 350 | 6 | 350 | 17 |

LLOQ was defined as the sample in reference standard curve (Tau 441 in artificial CSF) where mean bias of the measured concentration value relative to the known Tau 441 concentration in reference standard was ≤ 25%. CV for all samples was ≤ 20%.
