# Supplementary material for: An immuno-enrichment free, validated quantification of tau protein in human CSF by LC-MS/MS
Source: PLoS One. 2022 Jun 2;17(6):e0269157. doi: 10.1371/journal.pone.0269157 (PMC9162344; doi:10.1371/journal.pone.0269157)
Supplement: S5 Table — (DOCX) [file pone.0269157.s005.docx]

**S5 Table.** Forced Oxidation Optimization, using the protocol established by Barthelemy et al. [17] (Fig 1A).

| (25-44) DQGGYT**M**HQDQEGDTDAGLK | | 722.63 m/z  Peak Area | 727.92 m/z  Peak Area | 733.30 m/z  Peak Area | 722.63 m/z  Peak Area % | 727.92 m/z  Peak Area % | 733.30 m/z  Peak Area % |
| --- | --- | --- | --- | --- | --- | --- | --- |
| Oxidation Status | Time in autosampler (hr) |  |  |  |  |  |  |
| Forced | 0 | 2.62E+04 | 4.95E+06 | 6.93E+05 | 0.46 | 87.32 | 12.22 |
| Forced | 24 | 6.97E+03 | 8.87E+06 | 5.14E+05 | 0.07 | 94.45 | 5.48 |
| Forced | 96 | 0.00E+00 | 5.11E+06 | 3.42E+05 | 0 | 93.73 | 6.27 |
| None | 0 | 2.22E+07 | 2.31E+06 | 0.00E+00 | 90.56 | 9.44 | 0 |
| None | 24 | 2.67E+07 | 3.00E+06 | 4.80E+03 | 89.90 | 10.08 | 0.02 |
| None | 72 | 1.64E+07 | 2.41E+06 | 4.69E+03 | 87.17 | 12.80 | 0.03 |
| (243-254) LQTAPVP**M**PDLK | | 655.36 m/z  Peak Area | 663.36 m/z  Peak Area | 671.36 m/z  Peak Area | 655.36 m/z  Peak Area % | 663.36 m/z  Peak Area % | 671.36 m/z  Peak Area % |
| Forced | 0 | 3.17E+03 | 2.51E+07 | 3.01E+06 | 0.01 | 89.30 | 10.69 |
| Forced | 24 | 3.12E+04 | 3.90E+07 | 3.31E+06 | 0.07 | 92.10 | 7.83 |
| Forced | 96 | 0.00E+00 | 1.95E+07 | 1.69E+06 | 0 | 92.01 | 7.99 |
| None | 0 | 4.08E+07 | 6.53E+06 | 7.56E+03 | 86.20 | 13.78 | 0.02 |
| None | 24 | 6.22E+07 | 1.19E+07 | 2.09E+05 | 83.72 | 16.00 | 0.28 |
| None | 72 | 1.21E+07 | 1.63E+06 | 3.14E+04 | 87.89 | 11.88 | 0.23 |

Peak area % was determined by dividing the peak area for a single precursor across the sum of the peak area across all precursor ions analyzed. 722.63 , 655.36 m/z = unoxidized methionine. 727.92, 663.36 m/z = monoxidized methionine (+16 Da). 733.30, 671.36 m/z = deoxidized methionine (+32 Da).
